# Isolation of a Pericentromeric Satellite DNA Family in *Chnootriba argus* (*Henosepilachna argus*) with an Unusual Short Repeat Unit (TTAAAA) for Beetles

**DOI:** 10.3390/insects10090306

**Published:** 2019-09-19

**Authors:** Pablo Mora, Jesús Vela, Areli Ruiz-Mena, Teresa Palomeque, Pedro Lorite

**Affiliations:** Department of Experimental Biology, Genetic Area, University of Jaén, 23071 Jaén, Spain; pmora@ujaen.es (P.M.); jvela@ujaen.es (J.V.); armena@ujaen.es (A.R.-M.); tpalome@ujaen.es (T.P.)

**Keywords:** Coleoptera, Coccinellidae, bryony ladybird, satellite DNA, C0t-1 DNA, heterochromatin, in situ hybridization

## Abstract

Ladybird beetles (Coccinellidae) are one of the largest groups of beetles. Among them, some species are of economic interest since they can act as a biological control for some agricultural pests whereas other species are phytophagous and can damage crops. *Chnootriba argus* (Coccinellidae, Epilachnini) has large heterochromatic pericentromeric blocks on all chromosomes, including both sexual chromosomes. Classical digestion of total genomic DNA using restriction endonucleases failed to find the satellite DNA located on these heterochromatic regions. Cloning of C0t-1 DNA resulted in the isolation of a repetitive DNA with a repeat unit of six base pairs, TTAAAA. The amount of TTAAAA repeat in the *C. argus* genome was about 20%. Fluorescence in situ hybridization (FISH) analysis and digestion of chromosomes with the endonuclease *Tru*9I revealed that this repetitive DNA could be considered as the putative pericentromeric satellite DNA (satDNA) in this species. The presence of this satellite DNA was tested in other species of the tribe Epilachnini and it is also present in *Epilachna paenulata.* In both species, the TTAAAA repeat seems to be the main satellite DNA and it is located on the pericentromeric region on all chromosomes. The size of this satDNA, which has only six base pairs is unusual in Coleoptera satellite DNAs, where satDNAs usually have repeat units of a much larger size. Southern hybridization and FISH proved that this satDNA is conserved in some Epilachnini species but not in others. This result is in concordance with the controversial phylogenetic relationships among the genera of the tribe Epilachnini, where the limits between genera are unclear.

## 1. Introduction

Ladybugs (Coccinellidae) are one of the most important, large group of insects with almost 6000 species found all over the world [1]. The majority of these species are considered as beneficial due to their alimentation. They are predators of aphids, and hence, they can be used as a biological control against crop pests. However, all species of the Epilachnini tribe are phytophagous [2] including the bryony ladybird *Chnootriba argus* (*Henosepilachna argus* Geoffroy, 1762). This species feeds mainly on squirting cucumber (*Ecballium elaterium*), but also on other cucurbitaceous or solanaceous plants [3]. Although these insects can damage the host plant, it is believed that they may be important in pollen transfer in cucurbit crops [4].

Tandem repetitive DNAs or satellite DNA (satDNA) and transposable elements (TEs) are the main components of eukaryotic genomes. SatDNAs are mainly located in the centromeric and telomeric heterochromatic regions of the chromosomes but can also be found in the interstitial regions of the chromosomes [5,6,7,8]. Most eukaryotic centromeres are composed of repetitive DNA such as satDNAs, hence, they are sometimes identified as “satellite centromeres” [5]. Pericentromeric satDNAs are indispensable sequences since they stabilize the interactions between DNA, and DNA binding proteins maintain the heterochromatin architecture and play an important role in pairing and segregation in meiosis and mitosis [6,7,8]. The wide range of variation found in the eukaryotic genome size is mainly attributed to differences in the amount of repetitive DNA sequences [7]. Many satDNA families are conserved in a taxonomic group, allowing the use of these repetitive sequences in phylogenetic analyses and in taxonomic studies [7].

In the tribe Epilachnini, the genome size is known for only five species of the *Henosepilachna* genus; these range in size from 0.66 to 1.42 pg [9]. It has been suggested that the increase in the genome size in this species complex is not due to the change in the ploidy since there are small differences in the chromosome number among them [10]. Similar results were observed when all Coccinellidae species were analyzed. The genome size is only known for less than 40 Coccinellidae species [10]. However, there is a wide variation in size with some species with very small genome sizes (0.19 pg) and other species with a genome that is nine times larger than this (1.7 pg). The chromosome number in the 201 analyzed species of Coccinellidae is a little variable, 2n = 14–24, with 2n = 20 being the most common chromosome number [11]. These results suggest that the evolution and diversification of Coccinellidae has been accompanied by important changes in genome size, probably due to variations in the amount of repetitive sequences as satDNA or transposable elements.

The ladybird beetle *Chnootriba argus* is characterized by the presence of large heterochromatic blocks on all chromosomes [12]. Usually, the isolation of the satDNA is carried out by digestion of total genomic DNA using restriction endonucleases. In a previous study, a subtelomeric satDNA family was isolated in *C. argus* by digestion with the restriction endonuclease *Msp*I [13]. However, digestions with a great number of restriction endonucleases failed to find the satDNA located on the heterochromatic regions of this species [13]. Waring and Britten [14] developed a C0t-DNA analysis based on the ability of denatured DNA to re-nature into double strand DNA due to the pair base complementarity. High values of C0t have single copies whereas low values of C0t are repetitive sequences. By using C0t-1DNA, repetitive sequences (such as satDNAs) can be isolated avoiding the single copy sequences [15]. Thus, C0t DNA isolation is a powerful tool to isolate the repetitive sequences that are present in a genome. The use of the C0t-1 DNA fraction as a probe for fluorescence in situ hybridization (FISH) is a useful tool to analyze the organization and distribution of the heterochromatic DNA [16,17].

In this study, one satDNA family was isolated from *C. argus* by using the C0t-1 technique. This satDNA is located on the heterochromatic pericentromeric regions on all chromosomes. The amount of this satDNA was quantified in relation to the total genomic DNA. Based on its abundance, it probably represents the main satDNA family in this species. The most interesting result was the small size of the repeat unit. This satDNA was organized by repeating the sequence TTAAAA. Southern hybridization and fluorescence in situ hybridization proved that this satDNA are conserved in certain Epilachnini species but not in others. This result is in concordance with the controversial phylogenetic relationships among all of the genera of Epilachnini tribe, with unclear limits of the genera [18,19].

## 2. Materials and Methods

### 2.1. Materials

This study was carried out with wild *Chnootriba argus* collected by the authors on *Ecballium elaterium* plants on the campus of the University of Jaén, Spain (37.79 N, –3.78 W). Other Epilachnini species were also used: *Henosepilachna vigintioctomaculata* Motschulsky, 1857 (Hokkaido, Japan), *Henosepilachna septima* Dieke, 1947 (Islamabad, Pakistan), *Epilachna paenulata* Germar, 1824 (Montevideo, Uruguay) and *Diekeana admirabilis* (*Epilachna admirabilis* Crotch, 1874) (Hadano, Japan). No specific permission was required for the insect collection performed in this work, and no endangered or protected species were involved. Samples were killed and stored in −20 °C absolute ethanol until DNA extraction.

### 2.2. Extraction of Genomic DNA and Isolation of Repetitive C0t-1 DNA Library

Genomic DNA was isolated using a NucleoSpin Tissue kit (Macherey-Nagel GmbH & Co., Düren, Germany), following the manufacturer´s instructions. DNA concentration and purity was estimated by measuring the absorbance at 260 nm and 320 nm using a NanoDrop Lite spectrophotometer (Thermo Fisher Scientific, Waltham, MA, USA). *C. argus* genomic DNA shearing and C0t-1 DNA isolation were performed following the method described by Zwick et al. [15] with some modifications. The genomic DNA from *C. argus* (100–500 ng/μL in 0.3 M NaCl) was autoclaved for 30 min at 1.4 atm and 120 °C. Then an electrophoresis was carried out to check the expected size of DNA (fragments between 100 and 1000 bp). Three samples of 100 μL each were denatured at 95 °C for 10 min (M1, M2 and M3). The M1 sample was denatured and immediately put into ice for 10 s and treated with S1 nuclease [20,21] for 8 min at 37 °C. After denaturalization, M2 and M3 samples were put into ice for 10 s and renatured at 65 °C for 1 and 3 min, respectively, and then treated with S1 nuclease. DNA from the three samples was extracted with phenol-chloroform [22] and stored at −80 °C. The DNA from the M1, M2 and M3 samples was inserted into the pUC19 vector *Sma*I site. The ligation mix was used to transform *Escherichia coli* DH5α (Zymo Research, Tustin, CA, USA) competent cells that allowed the blue-white selection of recombinant plasmids using LB/ampicillin/IPTG/X-Gal plates.

A portion of the DNA from the M1, M2 and M3 samples was digoxigenin-labeled (DIG-11dUTP) by random priming with the DIG system (Roche Diagnostics GmbH, Germany) and used as hybridization probes for plasmids screening. Recombinant plasmids yielding positive hybridization signals were directly sequenced on both strands using the universal primers SP6 and T7 by a dideoxy sequencing method.

### 2.3. Chromosome Preparations, Probe Preparation and Fluorescence In Situ Hybridization

Chromosome spreads were obtained from adult male gonads following the method described by Lorite et al. [23]. The DNA probe for FISH was generated by a polymerase chain reaction (PCR) as described previously by Lorite et al. [24], using (TTAAAA)_4_ and (AATTTT)_6_ oligonucleotides as primers without template. These primers, when mixed, annealed to two stable double-stranded DNA forms with 3´and 5´ protruding ends. PCR generated fragments between 200 and 1000 bp fragments that were labelled with biotin-16-dUTP using the biotin nick translation kit (Roche Diagnostics GmbH, Mannheim, Germany). FISH was carried out following the procedure described by Lorite et al. [24] and Palomeque et al. [25] using the biotin labelled probe (2 ng probe/mL, 50% formamide). Fluorescence immunological detection was performed using the avidin-FITC/anti-avidin-biotin system without amplifications rounds. Slides were counterstained with propidium iodide and 4′-6-diamino-2-fenil-indol (DAPI).

### 2.4. Dot-Blot Hybridization

The quantification of TTAAAA repeat in the genome was estimated by dot-blot hybridization [26]. A series of dilutions of genomic DNA and plasmids containing the repetitive sequences were dot-blotted onto a charged nylon membrane and hybridized with a probe of the TTAAAA repeat labeled with DIG-11-dUTP. This probe was also generated by PCR as described above. Amplified DNA was labelled with DIG-11-dUTP using the DIG DNA Labeling Kit (Roche Diagnostics GmbH, Mannheim, Germany). Hybridization was performed at 60 °C using 20 ng of labeled probe/ml and a final wash in 0.5 × SSC at 60 °C. As negative controls, *Drosophila melanogaster* genomic DNA and pUC19 without any insert were used. The amount of satellite DNA in the genome was estimated by comparing the hybridization signals in the genomic DNA and plasmid dilutions. The presence of the TTAAAA repeat in other species was also tested by dot-blot hybridization. Several dilutions of genomic DNA for each species (1 µg to 62.5 ng) were loaded onto nylon membranes and hybridized with the TTAAAA probe.

### 2.5. Digestion with Restriction Endonucleases on Fixed Chromosomes

Chromosomes slides were treated according to the protocol described by Lorite et al. [27]. The slides were incubated with the restriction enzyme *Tru*9I (T/TAA). The incubation was performed in a moist chamber for 16 h with 20 U of the enzyme in 100 μL of the recommended buffer. Control preparations were incubated with the recommended buffer without the enzyme. The slides were mounted with Vectashield ® (Vector Laboratories, Burlingame, CA, USA) with DAPI and propidium iodide.

### 2.6. ND2 and 28S PCR Amplification and Phylogenetic Analysis

To assess the relative position of the species *Chnootriba argus* and *Epilachna paenulata* in relation to other Epilachnini species, we performed a phylogenetic analysis using previously published data [18]. In order to do so, we amplified the same sequences used by Katoh et al. [18]. Concretely two fragments of the *DN2* gene and the *28S* rRNA were used. The *ND2* gene fragment was amplified using the TM-J210 (5′-AATTAAGCTATTAGGTTCATACCC) and TW-N1284 (5′-TTAACTTTGAAGGTTAATAGTTT) primers designed by Simon et al. [28]. The *28S* rRNA fragment was amplified using the 28sf (5′-AAGGTAGCCAAATGCCTCATC) and 28sr (5′-AGTAGGGTAAAACTAACCT) primers [29]. PCRs were carried out in 25 μL reaction volumes, each containing 50 ng of genomic DNA, 10 pmol of each primer and 1 U of Taq polymerase. The PCR program used was 2 min at 92 °C and 35 cycles: 30 s at 92 °C, 60 s at 54 °C, 30 s at 72 °C, with a final extension of 4 min at 72 °C. PCR products were examined by 1% agarose gel electrophoresis, purified and sequenced on both strands with the same primers used for PCR amplification (GenBank accession no. MN190160–161, MN200099–100).

The phylogeny was conducted by employing the concatenated nucleotide sequences from both gene fragments. Multiple-sequence alignments were performed with ClustalW using BioEdit version 5.0.6 [30] and subsequently corrected by hand. The nucleotide substitution models were evaluated using MEGA version X [31]. The models with the lowest BIC scores (Bayesian Information Criterion) were considered the best to describe the substitution pattern. The phylogenetic relationships were analyzed using Maximum-likelihood (ML) methods and the GTR+G+I model with the MEGA X program. Bootstrap values for each branch were assessed from 1000 replicates.

Bayesian analyses were carried out using MrBayes version 3.1.2 [32]. Two independent runs were performed with four MCMC (Markov chain Monte Carlo) chains and run for 2,000,000 generations. Trees were sampled each 100th generations and a burn-in was set to 25% of samples. Convergence was considered to be reached when the average standard deviation of split frequencies was below 0.001. Finally, a 50% majority rule consensus tree was calculated from the obtained trees and the posterior probabilities were calculated using the command “sumt” in MrBayes.

## 3. Results and Discussion

A recent revision of the tribe Epilachnini placed the ladybird beetle *Henosepilachna argus* into the genus *Chnootriba* [33]. The chromosome number in this species is 2n = 18 with an Xy parachute sex chromosome system [12]. The Y chromosome was minute and for this reason it is often written with a lowercase letter. This chromosomal system is very common in Coleoptera and the nomenclature has already been used in classical cytogenetic studies [34]. Digestion of genomic DNA with the restriction endonuclease *Msp*I allowed the isolation of a satDNA family, named HargM with a repeat unit of about 650 bp. HargM satDNA is located on the subtelomeric regions of all chromosomes, with the exception of the long arm of the X chromosome [13]. PCR assays showed that the HargM satDNA was absent in other species of the Epilachnini tribe, specifically in *Henosepilachna vigintioctomaculata*, *Henosepilachna septima*, *Epilachna paenulata* and *Diekeana admirabilis*.

**Figure 1:**
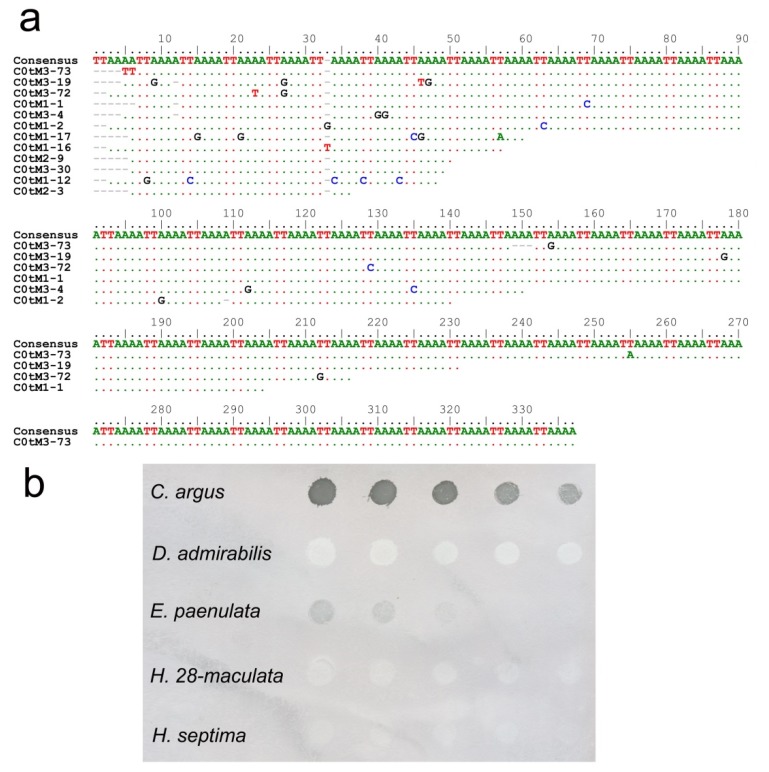
(**a**) Alignment of the sequences obtained by cloning of the C0t-1 DNA, showing that they were basically comprised of the repetition of the sequence TTAAAA. M1, M2 and M3 indicate the DNA sample used for cloning (see details in Materials and methods) (**b**) Dot-blot hybridization on total genomic DNA from *Chnootriba argus*, *Diekeana admirabilis*, *Epilachna paenulata*, *Henosepilachna vigintioctomaculata* and *Henosepilachna septima*. Different amount of genomic DNA (1 µg to 62.5 ng) were loaded into nylon membrane and hybridized with the TTAAAA repeat.

**Figure 2:**
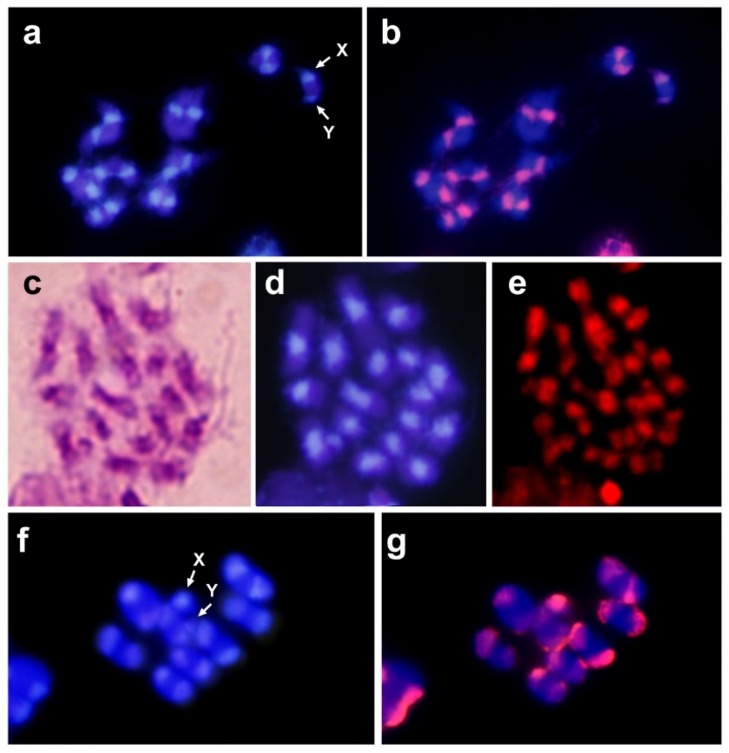
(**a**) Meiotic chromosomes of *Chnootriba argus* stained with DAPI and subsequently fluorescence in situ hybridization (FISH) with the TTAAAA probe (**b**). *Chnootriba argus* mitotic metaphase plate stained with Giemsa (**c**), DAPI (**d**) and digested with *Tru*9I and stained with propidium iodide (**e**). (**f**) Meiotic chromosomes of *Epilachna paenulata* stained with DAPI and subsequently, FISH with the TTAAAA probe (**g**). The arrows indicate the sex chromosomes (X and y).

C-banding of *C. argus* chromosomes showed the presence of large heterochromatic pericentromeric regions on all chromosomes, including the sexual pair. Staining with DAPI showed that the DNA located on these regions was A+T rich [12]. Since satDNA is the main component of the heterochromatin, another or other satDNA families must be present on those pericentromeric regions. In order to isolate the pericentromeric satDNA, a battery (more than 20) of restriction endonucleases was used to digest the genomic DNA. All tested endonucleases failed to find another repetitive DNA different to the 650 bp HargM satDNA. This method may have failed because of the low copy numbers of the satDNA or the inability to find an enzyme that cut the repeat unit [35]. Thus, analysis of C0t-1 DNA was used as an alternative strategy for satDNA isolation [36]. Fourteen clones were selected after screening of the *C. argus* C0t-1 library. Twelve of the 14 sequenced clones were composed of the tandem repetition of the hexanucleotide TTAAAA (Figure 1a). The size of the inserts varied between 30 and 329 nucleotides in length. The similarities among the obtained sequences were over 98%. Most changes in the sequence were due to punctual mutations as well as some indels of one nucleotide. Mutations appear to be spread randomly, and there are no signs of existence of any higher order repeat (HOR) in the analyzed sequences. Dot-blot analysis revealed that the amount of TTAAAA satDNA in the *C. argus* genome was about 20%, suggesting that the (TTAAAA)_n_ repeat is the main repetitive DNA in this species. Fluorescent in situ hybridization (FISH) was carried out to determine the location of the TTAAAA satDNA family. Hybridization positive signals appeared on the heterochromatic pericentromeric regions on all chromosomes, even the sexual ones (Figure 2b). These regions correspond with the heterochromatic regions of the chromosomes, which were positively stained with DAPI (Figure 2a).

In insects, the more common length of the repeat units of a satDNA family ranges between 100 and 400 bp [37]. In Coleoptera, the repeat units of the isolated satDNAs using conventional techniques are usually between these values [38], although, in some cases, the length is significantly higher, such as the 1.2 kb *Pst*I family of *Misolampus goudoti* [39] or the 1061-bp TBREV family of *Tribolium brevicornis* [40]. Among coleopteran species, the pericentromeric satDNA with the shorter repeat unit was the 109 bp LEDE-II family isolated in *Leptinotarsa decemlineata* [41]. Therefore, the TTAAAA satDNA isolated in *C. argus* was the pericentromeric satDNA with the shortest tandem repeat described to date in Coleoptera. In insects, it has been known for a long time that satDNAs with short repeat units exist in *Drosophila* species. In *Drosophila virilis* there are three families of satDNA that together represent about the 40–50% of the genomic DNA. The repeat units of these satellites are heptanucleotides that differ one from the other only by simple base-pair changes; ACAAACT (SatI), ATAAACT (SatII) and ACAAATT (SatIII) [42]. The heterochromatin in *Drosophila melanogaster* contains several families of short repeats (5–12 bp). These satDNA families are not present on all centromeres and are located in other chromosome regions [43]. In *Drosophila hydei,* the presence of a telomere-like satellite DNA that comprised 4% of the total genomic DNA was reported [44]. This satellite DNA has a repeat unit of 7 bp and it is located on the centromeric heterochromatin of the autosomes. 

Traditionally, tandem repeats have been classified into three categories according to the size of the repeat units: microsatellites (1–10 bp), minisatellites (> 10 bp) and satellite DNAs. It is generally considered that microsatellites and minisatellites are organized in short arrays (< 1 kb) and are scattered throughout the euchromatin. However, it is considered that satellite DNA (satDNA) is organized in long arrays of longer repeat units and located on the heterochromatic regions, mainly in the pericentromeric and subtelomeric regions [8]. The TTAAAA repetition of *C. argus* has the size of a microsatellite but its abundance and location are typical of satDNAs, according to the classical definition. This suggests that the classification in microsatellites, minisatellites and satDNA is likely to be artificial. This is not a new idea and it has been suggested by other authors such as Ruiz-Ruano et al. [45]. In the grasshopper *Locusta migratoria*, 62 satDNA families were characterized using Next Generation Sequencing (NGS) and bioinformatics tools [45]. The authors demonstrated that the three types of repeats showed similarities at genomic and cytological levels. The different satDNA families could be organized as short or long arrays shared in the euchromatin or accumulated in the heterochromatin, regardless of the size of the repetition unit. For example, in hemipteran species of the subfamily Triatominae, the vector species of the Chagas disease, GATA repeats are the only satDNA family that is shared on the Y chromosome in species of the tribe Triatomini [46]. On the other hand, a satDNA family with a repeat unit 1 kb in length (TinfSat04-1000) was located on the euchromatin region of *Triatoma infestans* [46]. A satDNA similar to TinfSat04-1000 was isolated in another triatomine species, *Rhodnius prolixus*, and showed a similar distribution [47]. There are already many similar examples in both animals and plants, mainly due to the application of NGS and the analysis of a whole collection of different satDNA families in a genome [48,49,50,51]. The new term “satellitome” includes all families of satellite DNA present in a genome [45,51].

Restriction endonucleases (REs) have been widely used to produce chromosome banding since these enzymes cut the DNA and the generated fragments are removed of fixed chromosomes [52,53,54]. The removal of DNA fragments results in a reduction in staining when DNA dyes are used, which produce chromosome banding by in situ digestion. The effect of REs on fixed chromosomes depends on the amount of target sequences in the chromosomes, and to a large extent, on the accessibility of DNA in chromatin [27,55]. Therefore, condensed regions such as heterochromatic regions are usually resistant to RE digestion. In spite of this, REs are able to digest heterochromatic regions when the target for the RE is conserved in the repeat units of the satellite DNA located in these regions [56,57]. We analyzed the effect of the *Tru*9I RE on fixed chromosomes of *C. argus* since a tandem of TTAAAA repeats contains many targets for *Tru*9I (T/TAA)—one every 6 base pairs in the absence of mutations. Digestion with this enzyme produced the almost total loss of staining in the heterochromatic regions on the chromosomes of *C. argus* (Figure 2e). This suggests that these heterochromatic regions could be composed mainly of TTAAAA repeats.

Dot-blot analysis was used to determinate the presence and the amount of the TTAAAA satDNA in other species of the Epilachnini tribe; *Henosepilachna vigintioctomaculata*, *Henosepilachna septima*, *Epilachna paenulata* and *Diekeana admirabilis* (Figure 1b). Dot-blot revealed that the TTAAAA satDNA was only present in *Epilachna paenulata*, although the amount of TTAAAA repetition in its genome was significantly lower than in *C. argus*. This species has a diploid chromosome number of 2n = 16 + Xy and as in *C. argus*, showed large blocks of heterochromatin in the pericentromeric regions on all chromosomes [58]. FISH using the TTAAAA repeat as a probe showed that in *E. paenulata*, this sequence, as in *C. argus*, was located on the heterochromatic regions of the autosomes and on the sexual pair Xy (Figure 2g).

**Figure 3:**
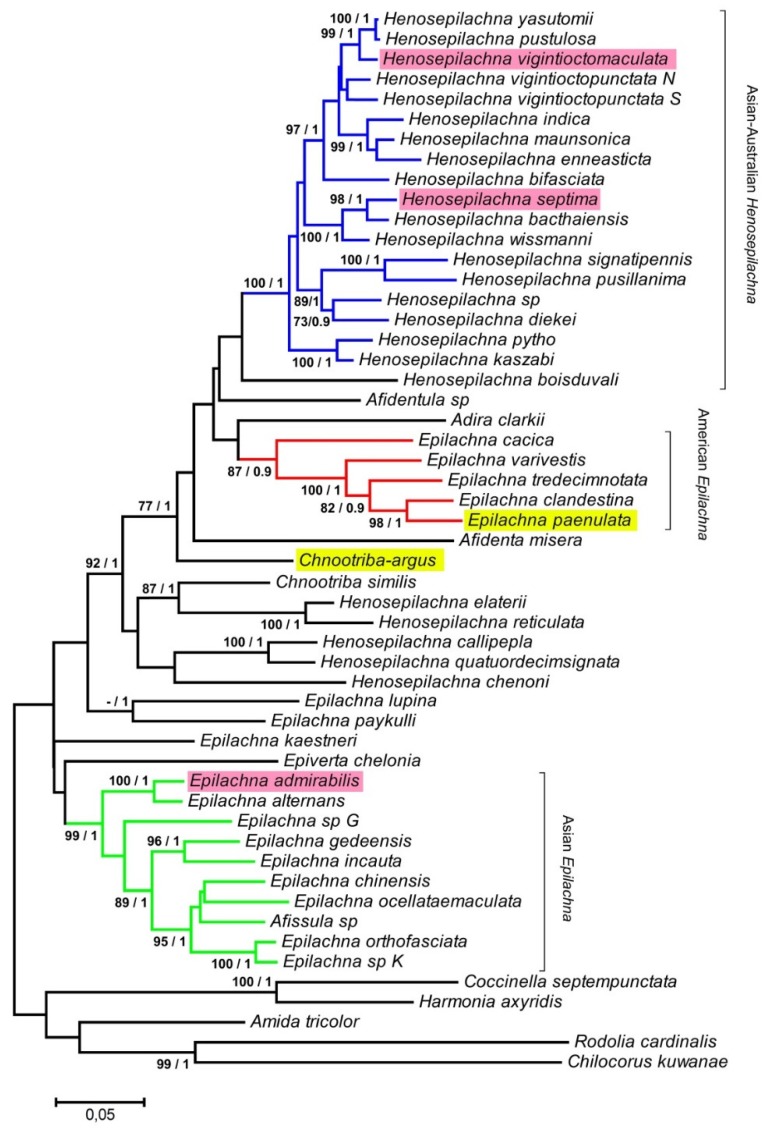
Phylogenetic tree using concatenated sequences of the *ND2* gene and the *28S* rDNA. The first number at nodes indicates the bootstrap values obtained in the maximum-likelihood analysis (only when higher than 70%) and the second indicates the posterior probability values in the Bayesian inference analysis (only when higher than 0.7). This tree was created using the data of a previous phylogeny [18] by adding the sequences of *Chnootriba argus* and *Epilachna paenulata*. All species appear with the name used in Katoh et al. [18], although the recent revision of the tribe Epilachnini places many of them in other genera [19,33]. Well-supported clades—Asian-Australian *Henosepilachna*, American *Epilachna* and Asian *Epilachna*—are highlighted with colors. Species with the TTAAAA satellite DNA are shaded in yellow whereas species shaded in pink lack this satellite DNA.

Although the Epilachnini tribe is recognized as a homogeneous group, the taxonomy of the different species within the group has been a controversial subject. Most species have traditionally been included within the genera *Epilachna* and *Henosepilachna*, making the limits between the two genera unclear. A phylogenetic study carried out by Katoh et al. [18] using *ND2* and *28S* datasets has shown that both genera are polyphyletic and that only three groups of species are in well-supported clades: Asian *Epilachna*, American *Epilachna* and Asian-Australian *Henosepilachna*. Neither *Chnootriba argus* nor *Epilachna paenulata* were included in this analysis. In order to assess the relative position of both species with other Epilachnini, *DN2* and *28S* sequences were amplified by PCR, and phylogenetic analyses were carried using the dataset previously included in Katoh et al. [18] (Figure 3). As expected, *E. paenulata* cluster with the other American *Epilachna* species and *C. argus* (formerly *Henosepilachna argus*) is placed near African *Henosepilachna*. The presence/absence of the TTAAAA satDNA is in concordance with the polyphyletic origin of *Epilachna* and *Henosepilachna*. A complete phylogenetic study with 153 species of Epilachnini using four DNA markers (*18S* and *28S* rRNA, *16S* and *COI* mtDNA) and a matrix of 104 morphological characters led to an intense revision of the generic classification of the tribe Epilachnini [19,33]. The genera *Henosepilachna* (new sense) now includes most of the Asian *Henosepilachna* (as *H. vigintioctomaculata* and *H. septima*, without TTAAAA satDNA). Other *Henosepilachna* species are included in other genera. For example, the Paleartic *Henosepilachna* species, as *H. argus* (with TTAAAA satDNA) are included in the genus *Chnootriba* as *C. argus*. The genera *Epilachna* (new sense) is now restricted to part of the New World *Epilachna* species as *E. paenulata* (with TTAAAA satDNA). The remaining *Epilachna* species have been placed in other genera as *Diekeana*, which includes *D. admirabilis* (without TTAAAA satDNA), formerly *Epilachna admirabilis*. There are not enough data to determine if the presence of the TTAAAA satDNA is a plesiomorphic character of the tribe Epilachnini and has been lost in some evolutionary lines, or if it is an apomorphic character that has appeared once or several times within the Epilachnini tribe. It would be interesting to carry out an extensive study of the TTAAAA satDNA in Epilachnini species to analyze its evolution.

## 4. Conclusions

The ladybird beetle *Chnootriba argus* (Coccinellidae, Epilachnini) is characterized by the presence of big heterochromatic pericentromeric blocks on all chromosomes. The use of a C0t-1 DNA library allowed the isolation of a satellite DNA family with a repeat unit of six base pairs, TTAAAA. FISH and digestion with restriction endonucleases showed that this satellite DNA is the main component of the heterochromatin in this species. TTAAAA satDNA is the pericentromeric satDNA with the shortest tandem repeat described to date in Coleoptera. This satellite DNA is probably the main satellite DNA in *C. argus* since it represents 20% of the genomic DNA. This satellite DNA is present in other species of the tribe Epilachnini but absent in others. The pattern of absence-presence is in concordance with the new taxonomic proposal for this tribe. The obtained information is necessary to understand the evolution of satDNA in Coleoptera as well as in other groups of organisms.
